# Concomitant disseminated histoplasmosis and disseminated tuberculosis after tumor necrosis factor inhibitor treatment: a case report

**DOI:** 10.1186/s12879-016-2097-7

**Published:** 2017-01-13

**Authors:** Juan E. Muñoz-Oca, Martha L. Villarreal Morales, Aracelis Nieves-Rodriguez, Lemuel Martínez-Bonilla

**Affiliations:** 1 JEMO: Family Medicine Residency Program, Manatí Medical Center, P.O Box 1142, Manatí, PR 00674 USA; 2 MLVM: Department of Medical Education, Manatí Medical Center, P.O Box 1142, Manatí, PR 00674 USA; 3 ANR: Department of Family Medicine, Manatí Medical Center, P.O Box 1142, Manatí, PR 00674 USA; 4 LMB: Department of Internal Medicine /Infectious diseases, Manatí Medical Center, P.O Box 1142, Manatí, PR 00674 USA

**Keywords:** Histoplasmosis, Tuberculosis, Co-infection, TNF-α inhibitors, Adalimumab, Case report

## Abstract

**Background:**

Tumor necrosis factor antagonist inhibitors have transformed the approach to patients with severe autoimmune conditions, such as rheumatoid arthritis. Although the therapy can be highly effective, TNF-α inhibitors are associated with an increased risk of opportunistic infections.

**Case presentation:**

Here, we report a case of concomitant disseminated histoplasmosis and tuberculosis in a 65-year-old female with rheumatoid arthritis treated with TNF-α inhibitor. Both conditions can be found in disseminated form in immunosuppressed hosts, but co-infection is rare with only a few cases having been reported, to our knowledge, all in HIV patients.

**Conclusions:**

This case posed a considerable challenge for diagnosis and treatment due to the unusual disseminated co-infection, the overlapping symptoms, and the interactions between medications.

## Background

Histoplasmosis is a systemic fungal disease acquired by inhalation of microconidia from the fungus *Histoplasma capsulatum.* Distribution is worldwide where it can be found in soil and in the droppings of birds and bats [1]. The spores are initially inhaled, reaching the alveoli, where they transform into budding yeast cells. Subsequently, the macrophages phagocytize the yeast although sometimes they are unable to eliminate it, which allows the yeast to multiply and spread via the lymphatic system. Once cell-mediated immunity develops, the new macrophages may eliminate the yeast or create a wall of histoplasma around it forming granulomas [1]. Tuberculosis (TB) is a global disease caused by *Mycobacterium tuberculosis.* It spreads from human to human via inhaled infectious particles through the lungs, and its resurgence has been associated with the human immunodeficiency virus (HIV) epidemic [2]. In 2014, the World Health Organization estimated that 9.6 million people had contracted TB and 1.5 million died. About 12% of the 9.6 million new TB cases were HIV-positive with 400 thousand deaths in the HIV-positive population [2]. In general, once the infected droplets are inhaled, the infection progresses in a similar fashion to histoplasmosis. Initially, the bacilli may multiply both in the alveoli and inside the macrophage until cell-mediated immunity develops. The infection can be controlled with the formation of a granuloma, where the CD4+ cells and Tumor necrosis factor alpha (TNF-α) macrophages are key factors for reactivation surveillance. However, new epidemiological and genetic data support the fact that in some instances, the body can successfully eradicate *M. tuberculosis* infection before an adaptive immune response develops, which is referred as early clearance [3]. It is important to point out that both Histoplasmosis and TB can be found in disseminated form in immunosuppressed hosts, yet co-infection is rare with only a few cases having been reported, to our knowledge all in HIV patients [4]. We are reporting a case of an immunosuppressed patient due to TNF-α inhibitor therapy, who was co-infected with Hisptolasmosis and TB, both in disseminated form.

## Case presentation

**Fig. 1:**
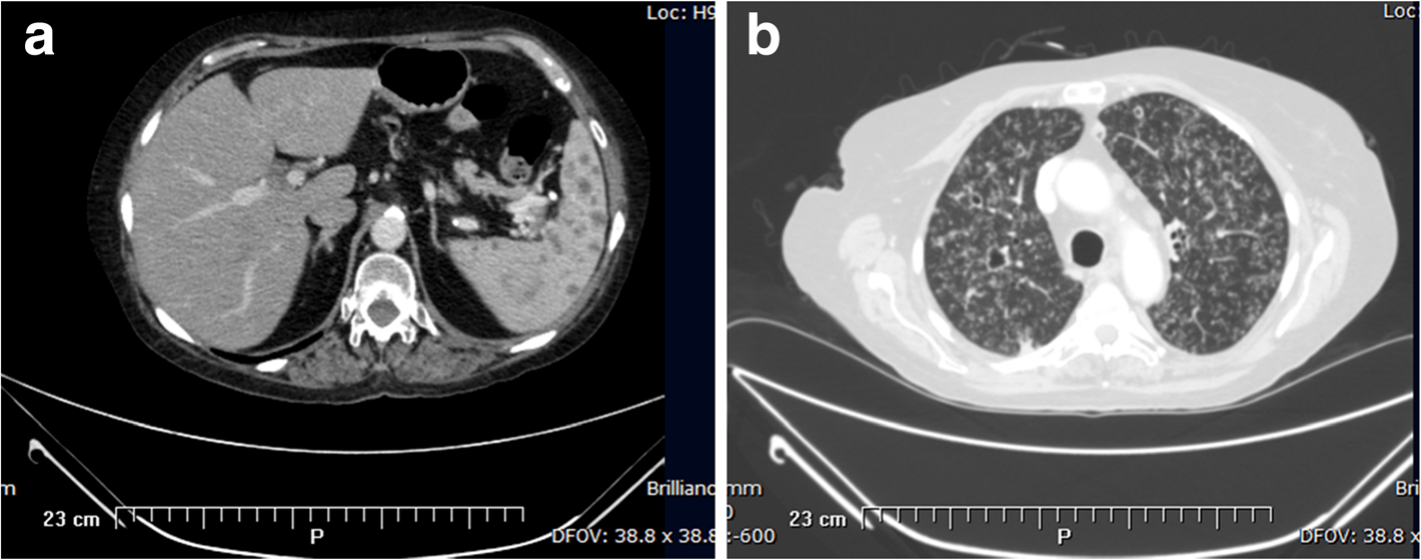
**a** Axial CT abdomen image demonstrates numerous hypo-attenuating lesions on spleen, almost replacing the normal parenchyma. **b** Axial CT chest image reveling reticulonodular infiltrates with tree in bud and cavitations

A 65-year-old female with rheumatoid arthritis treated with a TNF-α inhibitor (Adalimumab) presented to her primary care physician with episodes of malaise, dyspnea, fever, weight loss and pleural effusions on and off for six months. She underwent medical evaluation at another institution without obtaining a clear diagnosis. Since Adalimumab has also been associated with lymphoma and other cancers, the patient underwent an abdominal computerized tomography (CT) that revealed multiple hypoattenuating lesions in the spleen and retroperitoneal lymphadenopathy in the periportal, peripancreatic and mesenteric regions, some with necrotic appearance (Fig. 1a). A needle biopsy was done on the spleen, which showed multiple caseating granulomas and the use of special stains revealed the absence of acid fast bacilli but identified thin-walled yeast-like organisms with distinctive histopathological characteristics consistent with histoplasmosis. The patient was then referred to our institution and hospitalized due to disseminated Histoplasmosis.

At presentation the patient looked chronically ill, but was afebrile with stable vital signs. The physical examination was unremarkable except for her advanced interphalangeal joint deformities. Additional laboratory studies showed the white blood cell (WBC) count was 8,100/μL with a neutrophil count of 80.9%, the C-reactive protein level (CRP) was 10.20 mg/dL and ferritin was 1,827.34 ng/mL. The blood chemistry data revealed low protein levels (5.2 g/dL), low albumin levels (2.5 g/dL), no electrolyte imbalance and preserved renal and hepatic functions. An HIV test and Histoplasma serology came back negative. A Chest X-ray was negative.

During detailed history the patient reported a hobby of caring for pigeons. Further history revealed she had positive tuberculosis skin tests (TST) on multiple occasions, in addition to caring for a family member with tuberculosis. Despite this, the patient had never received treatment for latent tuberculosis as she had normal chest x-rays. Treatment for disseminated histoplasmosis was started with amphotericin B lipid formulation while further evaluation for tuberculosis was initiated. Reevaluation for tuberculosis revealed a positive TST at 30 mm. Additional lung evaluation was done with a CT scan which showed extensive bilateral centrilobular nodules with areas of cavitation in the upper lung lobes in a miliary pattern (Fig. 1b). The CT also revealed additional findings such as prevascular lymphadenopathy and parenchymal lesions. The patient’s sputum smear was evaluated due to concern of further Histoplasma spreading. It was negative for fungus by KOH/Calcofluor test yet was positive for acid fast bacilli (AFB). The culture identified by DNA probe was positive *for M. tuberculosis* complex, and subsequently positive for *M. tuberculosis* by specific Polymerase Chain Reaction (PCR). The bronchoalveolar lavage (BAL) smear and culture were also negative for fungus and positive for AFB and identified by DNA probe and PCR as *M. tuberculosis*. This allowed us to rule out Histoplasmosis as the cause of the miliary pattern observed on her CT, and to move towards a diagnosis of concomitant disseminated histoplasmosis and tuberculosis.

A four-drug regimen of isoniazid, rifampin, ethambutol and pyrazinamide for tuberculosis was started which was well tolerated. The patient did not tolerate the full cycle of amphotericin B, so transition to itraconazole was started before the 2 week induction regimen could be completed. Given a severe interaction between rifampin and itraconazole, rifampin was later replaced with levofloxacin. Though initially well tolerated, our patient developed a clinical respiratory deterioration that required mechanical ventilation. We suspected Immune Reconstitution Inflammatory Syndrome (IRIS), thus methylprednisolone was added to the treatment. However, after 72 h of treatment the medication was withdrawn due to upper gastrointestinal bleeding. The tuberculosis treatment was not withdrawn or altered further. After a week of supportive treatment the patient improved clinically and was extubated. After 35 days of intra-hospital care, the sputum samples cleared and the patient was discharged to continue outpatient treatment at the Puerto Rico Health Department Tuberculosis Clinics.

## Discussion

Disease-Modifying Anti-Rheumatic Drugs (DMARDs) have transformed the therapeutic approach to patients with severe autoimmune conditions, such as rheumatoid arthritis, psoriasis and inflammatory bowel disease. These therapies, including TNF-α inhibitors, although highly effective, can produce profound immune suppression and predispose patients to common upper respiratory tract infections as well as reactivation of cell mediated controlled infections [5]. Both histoplasma and tuberculosis have been increasingly reported with the use of TNF-α inhibitor. Consequently the Food and Drug Administration (FDA) has issued a “black box” warning in an attempt to increase awareness of this problem [5]. The Center for Disease Control and Prevention (CDC) recommends TB testing before initiating immunosuppressive therapy [6]. TST and Interferon-Gamma Release Assays (IGRAs) can be used to screen for latent TB and data suggests that IGRAs are not inferior to TST [6, 7]. An initial chest x-ray is also recommended for baseline measurement. These images may aid in identifying occult active infections like cavitations, which would require multi-drug therapy [6]. This case shows the importance of adhering to the current recommendations in which positive TST requires pharmacotherapy independently of the findings on the chest X-ray. This is especially important when patients are being evaluated for therapies that suppress cell immunity, since reactivation of latent TB is significantly higher in patients receiving TNF-α inhibitors. Previous studies have shown a five-fold increased risk of reactivation within the first 52 weeks after initiation of therapy [8].

In contrast, currently there are no definitive recommendations for histoplasmosis screening [9]. There was a Histoplasma skin test analog to TST but it is no longer commercially available due to cross-reactions with other fungi and low sensitivity in patients with disseminated infection [1]. Furthermore, no therapy recommendations have been studied to prevent histoplasmosis reactivation. This gap in knowledge is important, as the disease can disseminate to bone marrow, liver, spleen, adrenal glands, and meninges with a mortality rate of up to 20% [4]. The disseminated form of histoplasmosis is defined by the presence of an extra-pulmonary focus. In some instances, patients show obvious widespread dissemination but in others, focal disease in a single organ is the only manifestation of dissemination [9]. In our patient, the histoplasmosis dissemination was manifested by identification inside spleen granulomas, which were numerous and almost replacing the spleen parenchyma. She also showed intraabdominal lymphadenopathy and elevated ferritin levels none of which are specific for disseminated histoplasmosis but are highly suggestive of this diagnosis in the appropriate patient [9]. Attempts to culture *H. capsulatum* should always be pursued for a definitive diagnosis; however it is a fastidious, slow-growing organism. The disseminated form is hard to diagnose, especially in view of its difficulty to be isolated on routine laboratory cultures and the negative serology that often occurs in immunocompromised patients with disseminated infection who often fail to manifest an immune response [9]. Urine and serum antigens are usually used to make a diagnosis; however, these antigens assays are not always available. In our case, antigens were not accessible and the histopathologic diagnosis along with a strong clinical suspicion and epidemiologic features provided adequate information to diagnose and to start treatment.

Disseminated co-infection with these two agents is rare but it has been previously described in patients with HIV, making our case unique because our patient was HIV negative [4]. The co-infection poses a challenge for diagnosis because the diseases share many symptoms, such as fever, fatigue, night sweats, lymphadenopathy, cough, and dyspnea. Both diseases affect the lungs, with cavitation and infiltration identifiable through chest X-rays. They can both lead to pancytopenia, elevated liver enzymes and elevated acute phase reactants like ferritin. If a biopsy of the affected organ is available, then it is imperative to look for both fungal and acid-fast organisms. Co-infection treatment also represents a challenge because of the well-documented interaction between rifampin and itraconazole [10]. Rifampin is an inductor of the Cytochrome P450 and reduces the serum concentrations of itraconazole. Rifabutin is a common alternative to rifampin, but also decreases the serum concentration of itraconazole, which is the drug of choice for histoplasmosis [11]. Quinolones are effective against tuberculosis and its activity is also well documented [12]. In clinical trials with renal transplant patients, where rifampin decreases the drug levels of immunotherapies, the use of quinolones as an alternative has shown favorable treatment outcomes [2]. The daily dosing also blends into the tuberculosis regimen, simplifying the treatment and helping increase patient compliance.

The observed clinical respiratory deterioration of our patient was another challenge. It started shortly after the initiation of TB treatment. Paradoxical tuberculosis worsening syndrome, a form of IRIS, explains this clinical deterioration. The discontinuation of TNF-α therapy, 6 months before admission may also be an important contributing factor. The patient’s initial improvement followed by rapid deterioration is consistent with IRIS, the same as latter stabilization without alteration of treatment regimen [13]. The recognition of this paradoxical reaction was critical in our case to rule out treatment failure, drug-resistance, or toxicity. IRIS is commonly described after the initiation of highly active antiretroviral therapy in HIV-patients; however it has also been reported in non-HIV-patients following discontinuation of TNF-α therapy, corticosteroid withdrawal, recovery of neutropenia after chemotherapy, and disseminated TB treatment initiation [13, 14]. The use of steroids and/or nonsteroidal anti-inflammatory drugs (NSAIDs) and the optimal treatment duration in TB-IRIS remains a controversial issue [13]. When steroids are considered for the treatment of TB-IRIS, then the usual course is 4–6 weeks. But in our case the development of gastrointestinal bleeding while on mechanical ventilation led us to the early discontinuation of steroids. The continuation of anti-tuberculosis treatment without steroids or NSAIDs along with aggressive supportive care led to improvement of our patient.

## Conclusion

Opportunistic infections should be highly suspected in immunosuppressed patients on medications like TNF-α inhibitors or corticosteroids. Strict adherence to the tuberculosis screening and treatment guidelines is recommended. Even though the co-infection of histoplasmosis and tuberculosis is rare, it should be suspected and assessed.

## Abbreviations

AFB: Acid fast bacilli
BAL: Bronchoalveolar lavage
CDC: Center for Disease Control and Prevention
CRP: C-reactive protein level
CT: Computerized tomography
DMARDs: Disease-Modifying Anti-Rheumatic Drugs
FDA: Food and Drug Administration
HIV: Human immunodeficiency virus
IGRAs: Interferon-Gamma Release Assays
IRIS: Immune Reconstitution Inflammatory Syndrome
NSAIDs: Nonsteroidal anti-inflammatory drugs
PCR: Polymerase Chain Reaction
TB: Tuberculosis
TNF-α: Tumor necrosis factor antagonist
TST: Tuberculosis skin test
WBC: White blood cell
